# Neuroprotective Effects of N-methyl-(2S, 4R)-trans-4-hydroxy-L-proline (NMP) against Amyloid-β-Induced Alzheimer’s Disease Mouse Model

**DOI:** 10.3390/nu15234986

**Published:** 2023-12-01

**Authors:** Jawad Ali, Amjad Khan, Jun Sung Park, Muhammad Tahir, Waqas Ahmad, Kyonghwan Choe, Myeong Ok Kim

**Affiliations:** 1 Division of Life Science and Applied Life Science (BK21 FOUR), College of Natural Sciences, Gyeongsang National University, Jinju 52828, Republic of Korea; jawadali666@gnu.ac.kr (J.A.); amjadkhan@gnu.ac.kr (A.K.); jsp@gnu.ac.kr (J.S.P.); muhammadtahir30@gnu.ac.kr (M.T.); waqasahmad@gnu.ac.kr (W.A.); k.choe@gnu.ac.kr (K.C.); 2 Department of Psychiatry and Neuropsychology, School for Mental Health and Neuroscience (MHeNs), Maastricht University, 6229 ER Maastricht, The Netherlands; 3 Alz-Dementia Korea Co., Jinju 52828, Republic of Korea

**Keywords:** Alzheimer’s disease, amyloid beta (Aβ_1–42_), N-methyl-(2S, 4R)-Trans-4-hydroxy-L-proline (NMP), oxidative stress, neuroinflammation, neuroprotection

## Abstract

Alzheimer’s disease (AD), is a progressive neurodegenerative disorder that involves the deposition of β-amyloid plaques and the clinical symptoms of confusion, memory loss, and cognitive dysfunction. Despite enormous progress in the field, no curative treatment is available. Therefore, the current study was designed to determine the neuroprotective effects of N-methyl-(2S, 4R)-Trans-4-hydroxy-L-proline (NMP) obtained from Sideroxylon obtusifolium, a Brazilian folk medicine with anti-inflammatory and anti-oxidative properties. Here, for the first time, we explored the neuroprotective role of NMP in the Aβ_1–42_-injected mouse model of AD. After acclimatization, a single intracerebroventricular injection of Aβ_1–42_ (5 µL/5 min/mouse) in C57BL/6N mice induced significant amyloidogenesis, reactive gliosis, oxidative stress, neuroinflammation, and synaptic and memory deficits. However, an intraperitoneal injection of NMP at a dose of (50 mg/kg/day) for three consecutive weeks remarkably decreased beta secretase1 (BACE-1) and Aβ, activated the astrocyte and microglia expression level as well as downstream inflammatory mediators such as pNF-ĸB, TNF-α, and IL-1β. NPM also strongly attenuated oxidative stress, as evaluated by the expression level of NRF2/HO-1, and synaptic failure, by improving the level of both the presynaptic (SNAP-25 and SYN) and postsynaptic (PSD-95 and SNAP-23) regions of the synapses in the cortexes and hippocampi of the Aβ_1–42_-injected mice, contributing to cognitive improvement in AD and improving the behavioral deficits displayed in the Morris water maze and Y-maze. Overall, our data suggest that NMP provides potent multifactorial effects, including the inhibition of amyloid plaques, oxidative stress, neuroinflammation, and cognitive deficits.

## 1. Introduction

Alzheimer’s disease (AD) stands as the most common cause of dementia, characterized by a gradual decline in cognitive abilities and memory. AD mainly affects older people and it is characterized by the deposition of β-amyloid plaques, neurofibrillary tangles, and the dysfunction of synaptic proteins, which leads to nerve cell death [1]. Aβ aggregates interfere with neuronal communication at the synapses; and may lead to cell death, while neurofibrillary tangles (NFTs) occlude nutrients as well as other essential molecules transported within neurons [2]. An elevated level of β-secretase (BACE-1) in neuronal tissue is recognized to facilitate Aβ production and the generation of amyloid plaques, which are observed in the brains of AD patients [3]. Both toxic Aβ peptides, as well as tau proteins, initiate an immune response through glial activation, which aims to phagocytize these toxic metabolites. But, when these toxic metabolites accumulate and outweigh their clearance by glia, this leads to neuroinflammation and neurodegeneration [4].

Another mechanism through which Aβ plaques provide a major contribution toward AD development, is the abnormal production of reactive oxygen species (ROS) which results in oxidative stress [5]. Previous research has shown that high levels of ROS surrounded the Aβ oligomers in the neuronal tissue of AD patients, indicating a prominent role in AD pathogenesis [6]. These free radicals chelate various biomolecules (e.g., proteins, lipids, as well as DNA), leading to neuroinflammation and neuronal cell apoptosis, which are the prominent hallmarks of AD [7]. To prevent the deleterious effects produced by oxidative stress, cells employ the nuclear factor erythroid 2-related factor (NRF2) along with its subsequent signaling pathway, which accelerate the production of numerous antioxidant enzymes (HO-1, glutathione (GSH), glutathione S-transferase (GST), etc.) [5,8]. Furthermore, there is crosstalk between AD and neuroinflammation, which is indicated by the overproduction of proinflammatory cytokines and the involvement of several protein kinases such as glycogen synthase kinase 3β, which control the nuclear factor kappa-light-chain-enhancer (pNF-ĸB) signaling pathway [9]. During the inflammatory processes, chronic pNF-ĸB activation leads to oxidative stress, enhanced cytokine production, and microglial and astrocyte chemotaxis toward that area, which in turn accelerates a process called reactive gliosis. Reactive gliosis not only provokes the pathogenesis of neuroinflammation but also causes the disruption of synaptic neurotransmission related to AD [10]. Moreover, synaptic dysfunction, defined as synaptic activity and the loss of synapses has higher correlations with cognitive dysfunction in AD. Both the disruption of synaptic activity and the loss of synapses occur at the early stages of the disease [11]. In various reports, considerable information has been provided for a strong relationship between Aβ peptide’s interference with synaptic proteins and hippocampal long-term potentiation (LTP), a memory storage synapse [12].

N-methyl-(2S, 4R)-Trans-4-hydroxy-L-proline (NMP) is a main component of Sideroxylon obtusifolium (Roem. & Schult.) T.D Penn which belongs to the Sapotaceae family [13]. The S. obtusifolium tree has the common name jungle plum, and its bark, leaves, and fruit (small berries) contain several chemical compounds, such as flavonoids, saponins, steroids, triterpenes, proanthocyanidins, leucoanthocyanidins, and sugars. Due to the importance of medicinal herbs in Brazil, both its bark and leaves are commercially used as folk medicine for anti-inflammatory purposes [14]. It has been proven that natural compounds have few adverse effects and a potential therapeutic value in clinical practice. The previously reported effects of this compound include anti-oxidant, anti-inflammatory, anti-nociceptive, as well as hypoglycemic activity [15]. In addition, the anti-convulsing effects of the L-proline derivative were evaluated in different models of PTZ- and Pilo-induced epilepsy in mice models [16]. Furthermore, the nanoemulsion of Sideroxylon obtusifolium is used to treat schistosomiasis [17]. Pharmacological studies of NMP with respect to neuroinflammation and neurodegeneration are very few. In our present research, we determined the neuroprotective effects of NMP by considering its anti-inflammatory, antioxidant, and synaptic properties in AD mouse models of neuroinflammation and neurodegeneration. We performed behavioral, biochemical, as well as immunohistochemical assays to determine the possible benefits of NMP, especially in mouse models of AD.

## 2. Materials and Methods

### 2.1. Chemicals

N-methyl-(2S, 4R)-Trans-4-hydroxy-L-proline (NMP) and the primary antibodies were purchased from Santa Cruz Biotechnology, (Dallas, TX, USA), while amyloid beta peptide (Aβ_1–42,_ catalog number RP10017) was purchased from GenScript. The primary antibodies used in the study are shown in Table 1.

### 2.2. Animals

Adult wild-type male mice (C57BL/6N, ages 8–10 weeks, weight 25–30 g) were purchased from Samtako Bio (Osan, Republic of Korea). The mice were acclimatized for one week in the animal house under standard conditions of room temperature 23–25 °C. The conditions also included maintaining a relative humidity of 60 ± 10%, 12 h light/dark cycle, and allowing mice unrestricted access to both food and clean drinking water throughout the study. Animal handling as well as all experimental procedures (treatment, behavioral, and surgical) were conducted based on the sanctioned regulations (Approval ID: 125) set forth by the Animal Ethical Committee (IACUC) within the Division of Applied Life Sciences, Department of Biology, Gyeongsang National University, South Korea. 

### 2.3. Experimental Design

To determine the effects of NMP in the AD mouse model, thirty-two mice were randomly separated into four groups (*n* = 8 mice/group). Group I (Control) received 0.9% normal saline; Group II (Negative control), Aβ_1–42_ (5 µL/5 min/mouse); Group III (Treatment control), Aβ_1–42_ + NMP (50 mg/kg for 3 weeks); and in Group IV (Sham), NMP alone (50 mg/kg for 3 weeks) was injected. Aβ_1–42_ peptide solution from human origin was prepared at a concentration of 1 mg/mL using isotonic saline. The peptide was then subjected to oligomerization by being kept at 37 °C continuously for four consecutive days [18]. Following that, the clustered Aβ_1–42_ peptide (5 µL/5 min/mouse) was precisely administered into the brain ventricles (i.c.v) through a stereotaxic procedure using a Hamilton micro syringe (−0.2 mm anteroposterior (AP), −2.4 mm dorsoventrally (DV), 1 mm medio lateral (ML), relative to the bregma). The process involved using a combined anesthesia dosage of 0.05 mL/100 g body weight for xylazine and 0.1 mL/kg body weight for ketamine.

**Figure 1:**
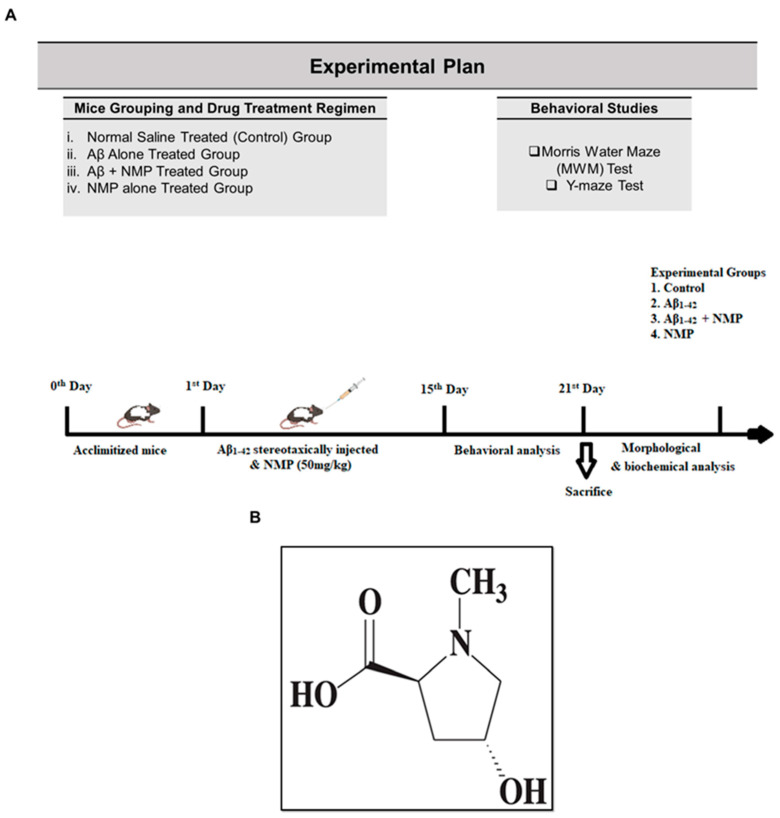
(**A**) Experimental plan for the current study of NMP against Aβ_1–42_-induced AD mice model. (**B**) Chemical structure of N-methyl-(2S, 4R)-Trans-4-hydroxy-L-proline.

The dosage regimen for control mice was 0.9% normal saline only, while the treatment control group was injected with NMP via the intraperitoneal route (50 mg/kg dissolved in normal saline) for 3 weeks. The NMP alone group was included to examine its cytotoxic, antioxidant, and neuroprotective effects in different biochemical assays (Figure 1).

### 2.4. Morris Water Maze

To investigate declarative learning and memory in laboratory mice, we conducted the MWM test after fourteen days of mice treatment according to the previous protocol, described with modifications [19]. This apparatus for the behavioral assessment consists of a circular water tank (of the following dimensions: diameter 100 cm, height 40 cm), filled with water (23 ± 1 °C) at a depth of 15.5 cm and made opaque by adding non-toxic white ink. At a height of 10 cm, a hidden transparent platform was placed in any of the quadrants of the tank (1 cm beneath the water surface). Each mouse underwent a series of five-day trials to find the location of the hidden platform with a starting point from the remaining three quadrants. If mice were unable to explore the hidden platform quadrant in 60 s, they were trained to the platform and remained there for 30 s. Each trial involved measuring the time taken to escape and the duration of time spent in the target quadrant. Subsequently, a probe test was conducted to assess memory consolidation, and the procedure was executed identically for each mouse, involving the removal of the hidden platform. In the probe test, the total time spent by each mice in the target quadrant, the number of crossings around platform, and the latency toward the platform were noted. The data was captured utilizing video-based motion tracking software (SMART 3.0, Panlab Harvard Apparatus, Bioscience Company, Holliston, MA, USA).

### 2.5. Y-Maze Task

To examine the spontaneous alteration of behavior with exploratory activity, we used a black painted wooden Y-maze with three arms (50 cm in length, 10 cm in width, and 20 cm in height) [20]. Briefly, each mouse completed one trial and was placed in the intersection of the three arms to freely explore the Y-maze for 8 min. Alterations and all arm entries were noted. The spontaneous alteration percentage (%) was calculated as (entries into three different arms consecutively/total number of arm entries-2) × 100. Spontaneous alteration with high percentage value was denoted as improved cognitive function and vice versa.

### 2.6. Extraction of Proteins from Mouse Brain

After 3 weeks of treatment, all the mice were numbed by anesthesia, killed, and the cortex and hippocampus of the brain were dissected and frozen on dry ice and kept at −80 °C for further processing. After that, PRO-PREP (iNtRON Biotechnology, Dallas Texas, MA, USA) solution was used for the homogenization of cortex as well as hippocampal tissues. Following that, the samples underwent centrifugation at a speed of 13,000 revolutions per minute (r.p.m) at 4 °C for 25 min. The tissue supernatants were gathered and preserved at a temperature of −80 °C for subsequent biochemical examinations. In the case of morphological analysis, the mice were anesthetized and transcardially perfused with 1X ice-cold PBS and 4% neutral buffer paraformaldehyde (NBP). After 48 h of NBP fixation, the brains were transferred to 20% sucrose solution for 72 h and fixed in O.C.T Compound, sliced into consistent 14 µm thick coronal sections using a microtome (Leica, cryostat CM, Nussloch, Germany), the sections were subsequently mounted onto ProbOn positively charged slides (Thermo Fisher, Waltham, MA, USA) for confocal microscopy using the thaw-mounting technique.

### 2.7. Western Blot Analysis

To evaluate the concentrations of various proteins linked to Alzheimer’s disease in both the cortex and hippocampus areas, Western blots were performed according to the previous protocol described [21]. Protein concentration in tissues was determined with the Bio-Rad protein assay kit (Bio-Rad laboratories, CA, USA). The gel electrophoresis utilized an equivalent amount of protein samples (25–30 µg), employing 4–12% BoltTM Mini Gels. A broad-range pre-stained protein ladder (GangNam-Stain^TM^, iNtRON Biotechnology) served as a control for molecular weights. Gels with different protein bands were placed on PVDF membranes. Then, these PVDF membranes were quenched for 1 h with skim milk (5% *w*/*v* skim milk in 1X TBST) in order to reduce the non-specific bindings of protein. After this, the membranes were left overnight at a temperature of 4 °C, incubated with primary antibodies diluted at a ratio of (1:1000 dilutions). The next day, after washing membranes with 1X TBST (10 min, 3 times), they were conjugated with corresponding secondary antibodies. ECL reagent (EzWestLumiOne, ATTO, Tokyo, Japan) was used for the identification of protein bands. Protein expression levels were captured on scanned X-ray films and were measured through densitometry analysis using computer-based ImageJ software (v. 150, NIH Bethesda, MD, USA).

### 2.8. Immunofluorescence Staining and Confocal Imaging

Immunofluorescent analysis was performed according to previous literature with few modifications [22]. Fourteen-micrometer coronal cryosections were taken from snap-frozen brains and dried overnight before staining. Slides holding cerebral tissue samples were washed with PBS twice for 10 min and blocking was performed with 5% normal goat serum for 1 h. Next, the slides were exposed to specific antibodies at a 1:100 dilution and kept at 4 °C for 24 h. The following day, the slides underwent PBS washing before being subjected to staining with anti-mouse/-goat/-rabbit secondary antibodies for a duration of 2 h at a dilution of 1:100. Afterward, the procedure involved exposure to 4′,6-diamidino-2-phenylindole (DAPI) for a duration of 10 min. Coverslips and a mounting medium (DAKO) were employed to encase the slides, and the observations of the images were conducted using a confocal laser microscope (FV3000 Operational Manual; Olympus, Tokyo, Japan). Confocal images were quantified and relative integrated density (sum of all the pixels within a specific region of the microscopic image and the mean gray value) via ImageJ software and graphs as well as statistical calculations were performed using GraphPad Prism software (ver. 8.0, San Diego, CA, USA).

### 2.9. Statistical Analysis

Western blot bands were scanned and analyzed via densitometry using ImageJ software. Results were expressed as the mean ± standard error of the mean (SEM). One-way analysis of variance (ANOVA) followed by Tukey’s multiple comparison test was used for different experimental groups. Behavioral data comprised 8 mice per group, whereas Western blot and confocal data consisted of 4 mice per group, respectively. These findings were indicative of three separate experiments. Graphs were generated and data were analyzed via GraphPad Prism software (ver. 8.0, San Diego, CA, USA). *p* values less than or equal to 0.05 were statistically significant. * *p* < 0.05, ** *p* < 0.01 indicate significant difference from Aβ_1–42_-treated group and ## *p* < 0.01 indicates significant difference from the vehicle-treated group.

## 3. Results

### 3.1. NMP Treatment Improved AD-like Pathology by Regulating the Level of BACE-1 and Aβ in Both the Cortex and Hippocampus of Aβ_1–42_-Induced AD Mice

**Figure 2:**
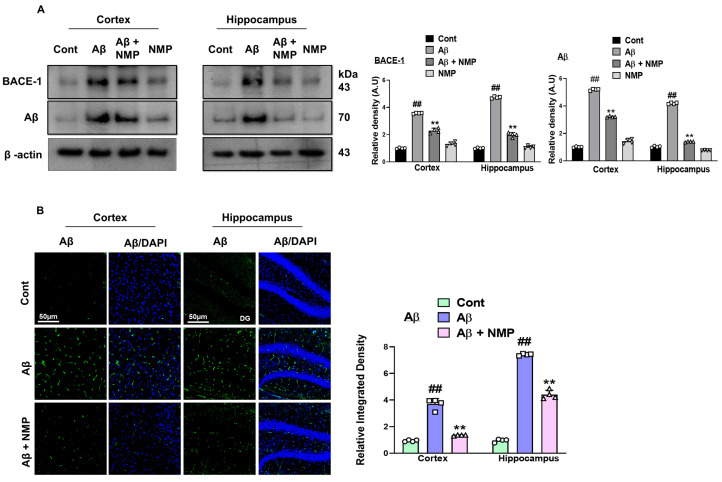
The impact of NMP on Alzheimer’s protein indicators within the brains of mice induced with Aβ_1–42_. (**A**) Immunoblot analyses and bar graphs depicting the levels of BACE-1 and Aβ expression in the cortex and hippocampus of mouse brains after the administration of Aβ_1–42_ and NMP. (**B**) Representative images and a corresponding bar graph showing the relative integrated density for Aβ in the cortical and hippocampal tissue (DG region) of mouse brains (*n* = 4 mice/group). Photomicrograph of (10X) magnification and inset scale bar is 50 µm. Band intensities were measured using ImageJ software (ver. 8.0, San Diego, CA, USA), and the distinctions were illustrated through a bar graph generated by GraphPad Prism. Beta-actin was utilized as a reference for loading. The mean ± S.E.M values for the indicated proteins are displayed as relative integrated density levels (*n* = 4 mice/group). ** *p* < 0.01 signify a notable distinction compared to the Aβ_1–42_-treated group, while ## *p* < 0.01 signifies a significant contrast from the vehicle-treated group.

The synthesis of Aβ occurs through the breakdown of APP, by the enzyme named β-site amyloid precursor protein cleaving enzyme 1 (BACE-1), which is central to AD development, and thus is a main therapeutic target [23]. Previous studies have shown that stereotaxic Aβ_1–42_ injection (i.c.v) into the ventricles induces amyloidogenesis by elevating the level of BACE-1 and Aβ in both the cortexes and hippocampi of mice [24]. To determine the protective effects of NMP against amyloid beta-like pathology, we performed an immunoblot assay for BACE-1 and Aβ in the cortical and hippocampal tissue of the experimental mice. The results obtained from our Western blot experiments revealed the increased levels of BACE-1 and Aβ expression in both the cortexes and hippocampi of mice treated with Aβ_1–42_, in comparison to the control mice. The mice treated with NMP (50 mg/kg for 3 weeks) exhibited a noticeable reduction in the levels of BACE-1 and Aβ expression within both the cortex and hippocampus of the Aβ_1–42_-treated mice (Figure 2A) (*p* < 0.05). To reinforce our Western blot findings, we conducted an immunofluorescence assay specifically targeting Aβ, which revealed the increased immunoreactivity of amyloid beta in the model group. However, the NMP treatment significantly downregulated the expression of Aβ in the cortexes and hippocampi of the Aβ_1–42_-treated mice (Figure 2B).

### 3.2. NMP treatment Improved Reactive Gliosis in AD Mice Brain

**Figure 3:**
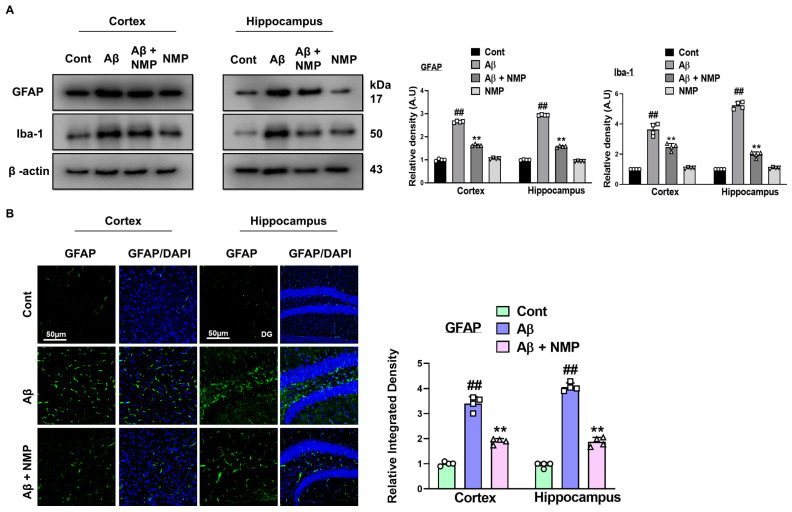
Effects of NMP on astrocytosis and microgliosis within the brains of mice injected with Aβ_1–42_. (**A**) Images of the scanned Western blot results and bar graph for indicated (GFAP and Iba-1) protein expression in the cortex and hippocampus of mice brain following Aβ_1–42_ and NMP treatment. The differences are shown in the bar graph. (**B**) Illustrative images along with an associated bar graph displaying relative integrated density of GFAP in the cortex and hippocampus (DG region) of mouse brains. Photomicrograph of (10X) magnification and inset scale bar is 50 µm. The information is displayed as the average value ± standard error of the mean (*n* = 4 mice per group). ** *p* < 0.01 vs. Aβ_1–42_-treated group and ## *p* < 0.01 vs. vehicle-treated group.

In neurodegenerative diseases such as AD, reactive gliosis and scarring, a common pathological process that occurs after brain injury, which involves the glial cells remaining in the affected area of the brain and secreting inhibitory factors to prevent neuronal cell growth [25]. The Glial fibrillary acidic protein (GFAP) and ionized calcium-binding adaptor molecule 1 (Iba-1) serve as established indicators used to distinguish inflammatory astrocytes and microglia, respectively. Our data showed that the Aβ_1–42_ injection prominently activated gliosis, as confirmed by the significantly increased expression level of GFAP and Iba-1 as compared to the normal control group. In contrast, the NMP-treated mice showed a remarkable decrease in the expression level of both GFAP and Iba-1 in the cortical and hippocampal tissues of the Aβ-treated mice, thus providing neuroprotective effects against Aβ_1–42_-induced gliosis (Figure 3A). The immunoblot results were additionally confirmed using an immunofluorescence assay, which showed that NMP prominently reversed the deleterious effects of Aβ on the glial cells in the cortexes and hippocampi of the Aβ_1–42_-treated mice, as shown by the relative density of GFAP (Figure 3B).

### 3.3. NMP Treatment Improved Oxidative Stress by Regulating the Level of NRF2 and HO-1 in Both Cortex and Hippocampus of Aβ_1–42_-Induced AD Mice

**Figure 4:**
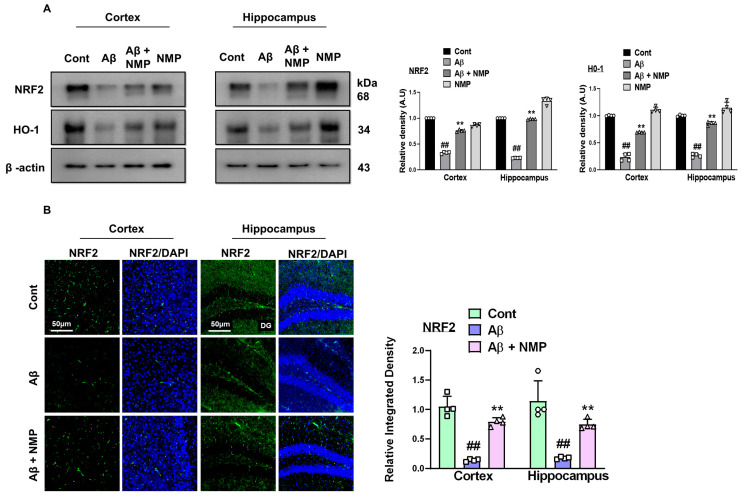
Effects of NMP on oxidative stress in the brain of Aβ_1–42_-induced mice. (**A**) Immunoblot analyses and bar graphs depicting the protein expression levels of NRF2 and HO-1 in the cortex and hippocampus of mouse brains after Aβ_1–42_ and NMP treatment. (**B**) Representative photographs and a corresponding bar graph showing relative integrated density of NRF2 in the cortex and hippocampus (DG region) of mouse brains. Photomicrograph of 10X magnification, and inset scale bar is 50 µm. Data are presented as the mean ± S.E.M (*n* = 4 mice/group). ** *p* < 0.01 vs. Aβ_1–42_-treated group and ## *p* < 0.01 vs. vehicle-treated group.

A failure in the redox balance leads to various neurodegenerative disorders including AD, and results in increased oxidative stress, damaging biomolecules, altered neuronal integrity, and neuronal cell death [26]. Through investigating the antioxidative process of NMP, we assessed the concentrations of nuclear factor erythroid 2-related factor 2 (NRF2) and its subsequent antioxidant enzyme, heme oxygenase-1 (HO-1), in the cortical and hippocampal tissues of mice treated with Aβ_1–42_. Our Western blot data revealed a decreased expression of NRF2 and HO-1 in Aβ_1–42_ -injected mice as compared to the normal control, whereas the NMP-treated mice showed increased the NRF2 and HO-1 protein expression in the brain tissue of the Aβ_1–42_ -treated mice. These results proved that NMP had the potential to trigger the upregulation of the NRF2 and HO-1 proteins in the cortical and hippocampal tissues of the Aβ_1–42_-treated mice (Figure 4A). In addition, through performing the immunofluorescence analysis, we confirmed that the Aβ_1–42_-injected mice showed decreased immunoreactivity of NRF2, while the NMP treatment significantly reversed these effects and elevated the immunofluorescence of NRF2 in the cortexes and hippocampi of the Aβ_1–42_-treated mice, suggesting its antioxidant potential (Figure 4B).

### 3.4. NMP Treatment Improved Neuroinflammation by Regulating the Level of pNF-ĸB and Its Downstream Targets in Aβ_1–42_-Induced AD Mice

**Figure 5:**
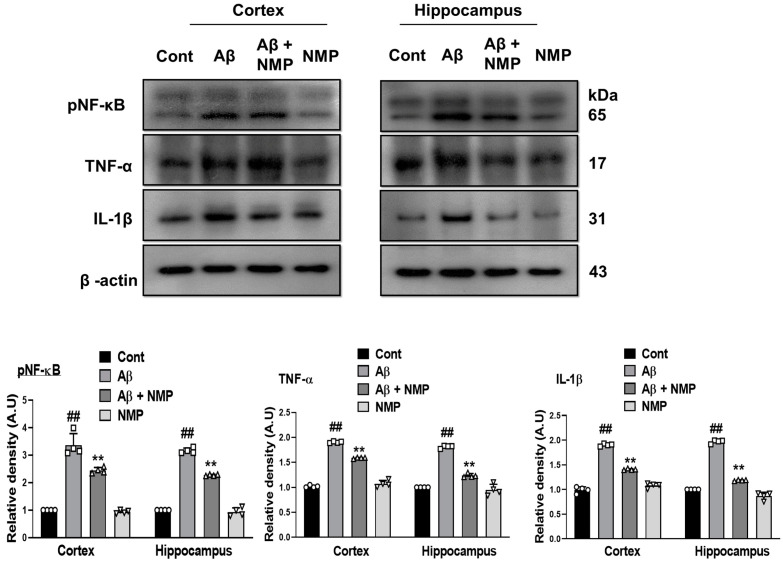
Effects of NMP on inflammatory cytokines as well as apoptotic marker in the brain of Aβ_1–42_-induced mice. The Western blot assessment and graphical representations indicating the protein expression levels of (pNF-κB, TNF-α, and IL-1β) in the cortexes and hippocampi of mouse brains after Aβ_1–42_ and NMP treatment. The bands were assessed and measured utilizing ImageJ software, and the differences are displayed in the bar chart. β-actin served as the standard for loading. The levels of relative density are presented in arbitrary units (A.U.) as the mean ± S.E.M for the specified proteins (*n* = 4 mice per group). ** *p* < 0.01 vs. Aβ_1–42_-treated group and ## *p* < 0.01 vs. vehicle-treated group.

Previous studies have indicated that the imbalance of the nuclear factor kappa-light-chain-enhancer (pNF-κB) is linked to various neurodegenerative conditions like Alzheimer’s disease, Parkinson’s disease, and Huntington’s disease [27]. In accordance with previous reports, our data showed a prominent increase in the level of pNF-κB and its downstream pro-inflammatory cytokines including tumor necrosis factor-alpha (TNF-α) and interleukin-1β (IL-1β) in the Aβ_1–42_-injected mice relative to the normal control, which were remarkably averted in the NMP-treated mice (Figure 5).

### 3.5. NMP Treatment Improved Synaptic Dysfunction by Regulating Synaptic Markers in Both Cortex and Hippocampus of Aβ_1–42_-Induced AD Mice

**Figure 6:**
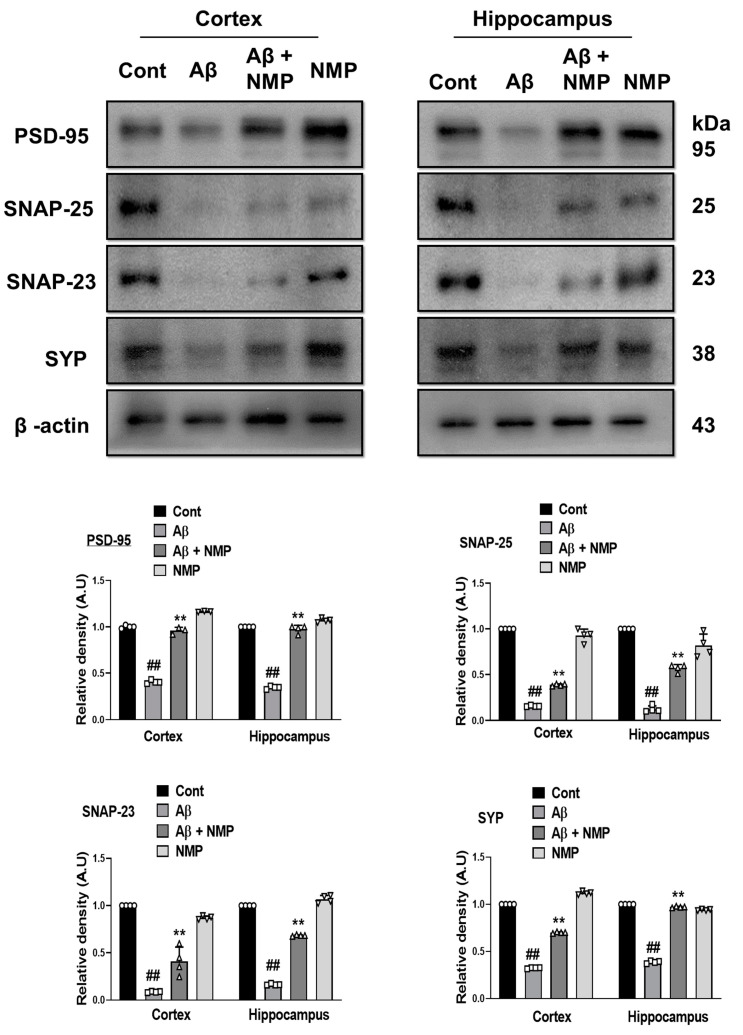
Effects of NMP on synaptic proteins in the brain of Aβ_1–42_-induced mice. Immunoblot analysis and bar graphs for the cortical and hippocampal protein expression levels of (PSD-95, SNAP-25, SNAP-23, and Synaptophysin) in the brains of mice, followed by Aβ_1–42_ and NMP administration. ImageJ software was used for the determination of band densities of these synaptic markers, while the differences are represented by the bar graph that was produced with GraphPad Prism 8 software. The data is displayed as the average ± standard error of the mean (*n* = 4 mice per group). ** *p* < 0.01 vs. Aβ_1–42_-treated group and ## *p* < 0.01 vs. vehicle-treated group. β-actin was used as a loading standard.

The major consequences of AD neuropathology are neuronal and synaptic dysfunction, which best correlates with cognitive and memory dysfunction [28]. To examine the protective effects of NMP against synaptic loss, we analyzed both presynaptic as well as postsynaptic markers such as post synaptic density protein (PSD-95), synaptosomal-associated protein 23 (SNAP-23), synaptosomal-associated protein 25 (SNAP-25), and synaptophysin (SYP), in the cortical and hippocampal tissues of the Aβ_1–42_-treated mice. According to our study, the level of synaptic proteins in the Aβ_1–42_-treated group declined relative to the control group. The NMP-treated mice reversed the synaptic protein loss caused by Aβ_1–42_ and significantly enhanced the level of both presynaptic and postsynaptic markers in the cortical and hippocampal tissues of the Aβ_1–42_-treated mice (Figure 6).

### 3.6. NMP Treatment Improved Aβ_1–42_-Induced Behavioral and Cognitive Deficits

**Figure 7:**
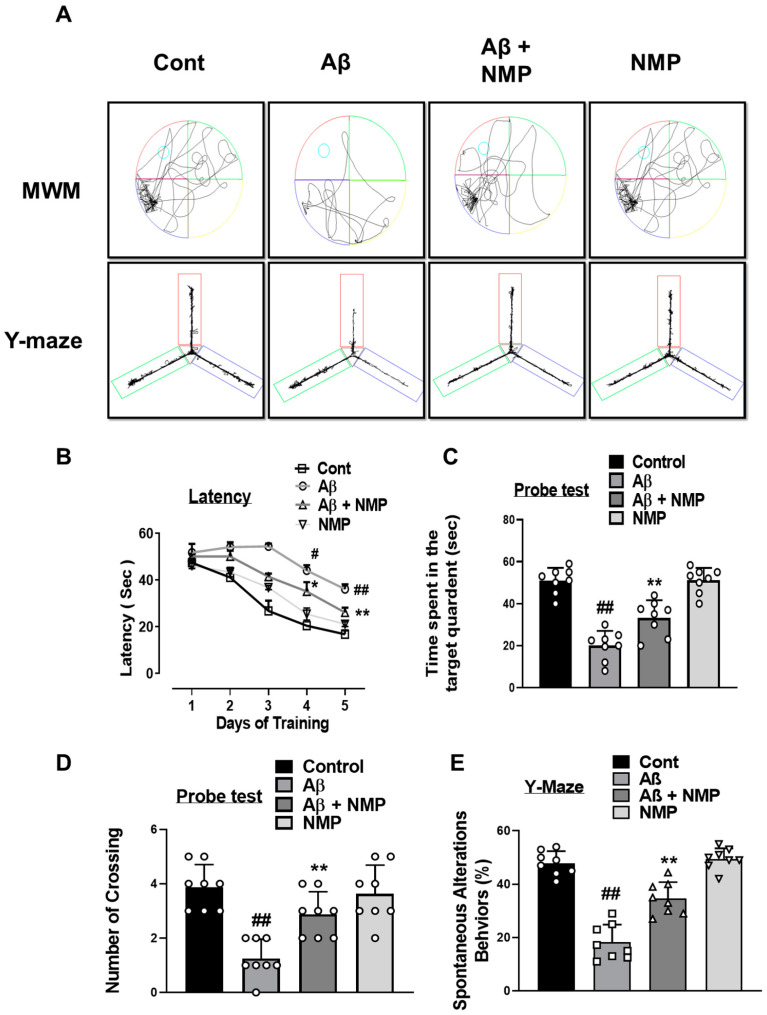
Effects of NMP on memory impairment and cognitive dysfunction in Aβ_1–42_-induced mice. (**A**) Images of the trajectory map in the MWM and Y-maze task. (**B**) Line graph showing mean escape latency during training days to reach the visible platform in the MWM task. (**C**) Time spent in the designated quadrant during the probe trial. (**D**) Number of crossings around platform during the probe trial. (**E**) Y-maze task for the measurement of spontaneous alteration behavior percentage in respective groups. The results are shown as the mean ± SEM (*n* = 8 mice/group). * *p* < 0.05, and ** *p* < 0.01 vs. Aβ_1–42_-treated group and # *p* < 0.05, and ## *p* < 0.01 vs. vehicle-treated group.

To investigate the protective effects of NMP on cognitive impairment and memory dysfunctions caused by Aβ_1–42_, we conducted a behavioral investigation involving the Morris water maze (MWM) and Y-maze assessment. During the MWM, we trained eight mice in each experimental group for five days to evaluate their learning abilities by practicing with a hidden platform. (Figure 7A). Our results showed that the escape latency over the course of the training prominently increased in the Aβ_1–42_-treated mice relative to the control mice. The NMP-treated mice showed a significant fall in the escape latency and improved cognitive function compared to the Aβ_1–42_-treated mice (Figure 7B). Next, after 5 days of training, we performed a probe test, which was more sensitive and specific to deficits in spatial learning. By removing the hidden platform in the probe trails, we noted a decrease in the number of crossings near the platform and a reduction in the duration spent in the target quadrant among the mice treated with Aβ_1–42_, an indication of Aβ_1–42_-induced cognitive dysfunctions. In contrast, the Aβ_1–42_ + NMP-treated mice showed a significantly increased acquisition of the crossing around the platform, and time spent in the target quadrant, as compared to the Aβ_1–42_-treated mice (Figure 7C,D). To validate the cognitive abilities of the mice with Alzheimer’s disease further, we conducted a Y-maze spontaneous alteration test to assess their spatial working memory, a type of short-term memory. The percentage of spontaneous alteration behaviors was evaluated in the form of a total number of arm entries, a measure of exploratory activity, and successive triplets. Our result indicated that, after Aβ_1–42_ injection, the percent of spontaneous alteration was lower in the Aβ_1–42_-treated mice than in the normal control mice, while NMP administration significantly enhanced the percent spontaneous alteration behavior, providing the evidence that the NMP treatment not only increased the exploration behavior but also mitigated the Aβ_1–42_-induced cognitive dysfunction (Figure 7A,E).

## 4. Discussion

Alzheimer’s disease (AD) stands as the most widely recognized and predominant cause of dementia. It represents a degenerative neurological condition that advances progressively, which is characterized by neuronal loss, the impairment of synapses, behavioral deficits, and, thus, eventually leads to the decline of both memory and thinking to carry out normal daily routines [29]. This study addressed the neuroprotective role of N-methyl-(2S, 4R)-Trans-4-hydroxy-L-proline (NMP) in vivo against an Aβ_1–42_-injected mouse model of AD. A single dose of amyloid beta (5 µL/5 mint/mice)-induced amyloidogenesis, memory impairment, oxidative stress, neuroinflammation, and neurodegeneration in the cortexes and hippocampi of adult mouse brains. Amyloid beta accumulation and aggregation in the neocortical regions of the human AD brain have been related to the onset of memory as well as cognitive dysfunction, while its clearance or reduction from the brain may be a principal approach for the prevention and treatment of AD [30,31]. The pathological propagation of amyloid beta in AD may occur through various functional brain networks [32]. The β-secretase enzyme, known as BACE-1, initiates the cleavage of the amyloid beta precursor protein (APP) in its ectodomain, leading to the production of various Aβ peptides of distinct lengths, among which is the disease-associated 42-amino acid peptide (Aβ_1–42_) [33]. Previous research has showed that the level of BACE-1 elevated by a two-fold in AD brain and its up-regulation may accelerate AD pathogenesis [34]. As BACE-1 is the rate-limiting enzyme for AD disease, considerable efforts were made to elucidate the significant role of NMP as a therapeutic agent that specifically targets BACE-1. To elucidate the protective effect of NMP against amyloid beta pathology, we conducted Western blot analysis and confocal microscopy for BACE-1 and Aβ in both cortical and hippocampal tissue homogenate, which showed the decreased expression levels of these protein markers, which is in accordance with previous literature [35].

During the progression of AD, the continuous stimulation of astrocytes and microglia in the brain can further promote AD pathology. This sustained and uncontrolled activation of astrocytes as well as microglia can lead to a state described as gliosis and has been implicated in AD pathogenesis [36,37]. The degree of reactive astrocytosis and microgliosis was evaluated through immunoblot and immunofluorescence assays. Our data also showed that both the Glial fibrillary acidic protein (GFAP) and ionized calcium-binding adaptor molecule 1 (Iba-1) markers in the neuronal tissue homogenate were significantly increased in AD, and the treatment with NMP prominently reduced the expression of these markers.

Multiple studies have proved that amyloid beta itself can initiate the production of reactive oxygen species (ROS), which in turn induces the occurrence of oxidative stress, thus leading to the pathogenesis of AD [38,39]. Many studies have proposed that the main therapeutic strategies lie in preventing the damage of biological macromolecules by oxidative stress, which is the inhibition of downstream signaling by ROS that leads to neuroinflammation and cell death signaling or increased antioxidant enzymes [39]. The scavenging of reactive oxygen species (O2− and H2O2) by the nuclear factor erythroid 2-related factor 2 and heme oxygenase-1 (NRF2/HO-1) signaling pathway was the primary method to achieve the goal of anti-oxidation, and a promising factor for improving the cognitive impairment seen in AD [40]. Previous reports have shown that NMP exhibits antioxidant behavior towards cytoplasm ROS accumulation and mitochondrial membrane depolarization caused by pilocarpine [41]. In this study, our Western blot analysis revealed that injecting Aβ_1–42_ led to a reduction in the expression of NRF2 and HO-1 in the cortexes and hippocampi of mice treated with amyloid beta. However, this decrease was notably reversed by the administration of NMP. Consistent with the previous study, the active gliosis and oxidative stress increased the expression level of nuclear factor kappa-light-chain-enhancer (pNF-κB) and its induced inflammatory cytokines tumor necrosis factor alpha (TNF-α) and interleukin-1β (IL-1β), which additionally enhanced the neuroinflammation and neurodegeneration [42]. Microglia and astrocytes are the major reservoirs of cytokines in AD and contribute to every aspect of neuroinflammation. An increased amyloid beta concentration in PSAPP and APPswe transgenic mice is associated with elevated concentrations of proinflammatory cytokines (TNF-α and IL-1β) [42]. In addition, microglial exposure to pre-aggregated Aβ_1–42_ increases the production of these cytokines, suggesting that the pathological accumulation of amyloid beta is a key mediator that directs neuroinflammation in AD [43,44]. A recent study showed that NMP acts as a potent anti-inflammatory agent and significantly decreases pNF-κB, TNF-α, and cyclooxygenase-2 (COX-2) expression in carrageenan-induced mouse paw edema [45]. The results of our present research also showed that NMP significantly attenuated the Aβ_1–42_-induced neuroinflammation by reducing the activation of pNF-κB and its downstream neuroinflammatory mediators.

Previous literature has shown that Aβ at very low concentrations enhances long-term potentiation (LTP) in the hippocampus and memory cognition, while the overproduction of Aβ leads to synaptic dysfunction [46]. Synaptic loss is a common hallmark of dementia, involving both specific presynaptic as well as postsynaptic proteins, which are postsynaptic density protein (PSD-95), synaptosomal-associated protein 23 (SNAP-23), synaptosomal-associated protein 25 (SNAP-25), and synaptophysin (SYP). These synaptic proteins are crucial for the exocytosis and endocytosis of various neurotransmitters and have been found to be altered in neurodegenerative disorders [47,48]. Our present data showed that the Aβ_1–42_ injection significantly downregulated both presynaptic and postsynaptic proteins; however, the administration of NMP notably reversed the decline in the synapses and increased the levels of proteins marking both presynaptic and postsynaptic activity. Similarly, our present data showed that the Aβ_1–42_ injection significantly reduced the memory cognitive function that was assessed via the Morris water maze (MWM) and Y-maze test. The MWM test showed a remarkable alteration in behavior, as indicated by the increased escape latency, decreased amount of time spent in the target quadrant, and fewer crossings around the platform during probe test in the Aβ_1–42_-injected mice. In addition, during the Y-maze test, we found that the NMP treatment enhanced the % spontaneous alteration behavior, which increased the spatial memory in the AD mice, consistent with previous research [49]. Interestingly, all these alterations were prominently ameliorated with the administration of NMP, providing evidence that NMP improved the memory impairments in AD mice.

**Figure 8:**
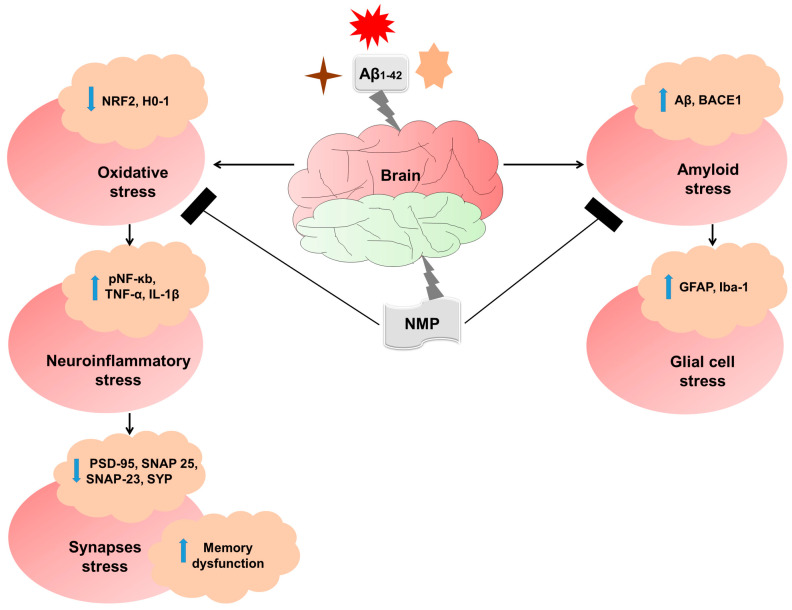
Graphical abstract showing the possible neuroprotective effects of NMP in Aβ_1–42_-induced mice. An accumulation of amyloid beta (Aβ_1–42_) stimulates amyloidogenesis, reactive gliosis, oxidative stress neuroinflammation, and synaptic and memory deficits. These effects are mitigated by NMP in neurodegenerative disorders by reducing the burden of amyloid plaques by reducing the amyloid plaques, gliosis, oxidative stress, as well as neuroinflammation by decreasing the expression level of BACE-1, Aβ, GFAP, Iba-1, ROS, and inflammatory cytokines, while increasing cognitive function by regulating synaptic markers.

Although our present research, up to large extent, covered various aspects and attained a large amount of progress in understanding the pathological consequences related to AD, there are some limitations to our study. The primary constraint lies in the relatively small sample size, emphasizing the necessity for a more extensive investigation to delineate the involvement of NMP in AD. Additionally, our model used an Aβ_1–42_-injected model to study AD, but this model might not develop as robustly amyloid plaques as 5xFAD or APP/PS1 transgenic mice. However, for our study, the Aβ_1–42_-injection model allowed us to determine reproducible phenotypes related to the amyloid beta accumulation as well as the temporal progression and its correlation with cognitive decline. Nevertheless, our study has shown that NMP treatment is neuroprotective against AD (Figure 8).

## 5. Conclusions

In conclusion, our findings suggested that NMP extracted from Sideroxylon obtusifolium reversed the amyloid beta-induced pathological consequences of Alzheimer’s disease, mainly via decreasing the amyloid burden, oxidative stress, synaptic/memory deficits, as well as the neuroinflammation and neurodegeneration in the cortical and hippocampal tissues of Aβ_1–42_-treated mice. Our present research fully supports the ability of NMP to improve AD-like pathological hallmarks and could be a safe, promising novel candidate to treat neurodegenerative disorders. In addition, preclinical trials are required to investigate the specificity of NMP in human subjects and its development in respect to neurodegenerative disorders related to AD and PD.

## Figures and Tables

**Table 1:** List of primary antibodies used in Western blot and immunofluorescence.

Protein Target	Source	Application	Dilution	Catalog Number	Manufacturer
BACE-1	Mouse	WB	1:1000	sc-33711	Santa Cruz Biotechnology
Aβ	Mouse	WB/IF	1:1000/1:100	sc-28365	Santa Cruz Biotechnology
Iba-1	Mouse	WB	1:1000	sc-39840	Santa Cruz Biotechnology
GFAP	Mouse	WB/IF	1:1000/1:100	sc-33673	Santa Cruz Biotechnology
pNF-ĸb	Mouse	WB	1:1000	sc-136548	Santa Cruz Biotechnology
TNF-α	Mouse	WB	1:1000	sc-52746	Santa Cruz Biotechnology
IL-1β	Mouse	WB	1:1000	sc-32294	Santa Cruz Biotechnology
NRF2	Mouse	WB/IF	1:1000/1:100	sc-365949	Santa Cruz Biotechnology
HO-1	Mouse	WB	1:1000	sc-136961	Santa Cruz Biotechnology
PSD-95	Mouse	WB	1:1000	sc-71933	Santa Cruz Biotechnology
SNAP-25	Mouse	WB	1:1000	sc-20038	Santa Cruz Biotechnology
SNAP-23	Mouse	WB	1:1000	sc-374215	Santa Cruz Biotechnology
Synaptophysin	Mouse	WB	1:1000	sc-17750	Santa Cruz Biotechnology

## Data Availability

Data is contained within the article.
